# Multimodal imaging needle combining optical coherence tomography and fluorescence for imaging of live breast cancer cells labeled with a fluorescent analog of tamoxifen

**DOI:** 10.1117/1.JBO.27.7.076004

**Published:** 2022-07-13

**Authors:** Loretta Scolaro, Dirk Lorenser, Bryden C. Quirk, Rodney W. Kirk, Louisa A. Ho, Elizabeth Thomas, Jiawen Li, Christobel M. Saunders, David D. Sampson, Rebecca O. Fuller, Robert A. McLaughlin

**Affiliations:** a The University of Adelaide, Australian Research Council Centre of Excellence for Nanoscale Biophotonics, Faculty of Health and Medical Sciences, Adelaide, South Australia, Australia; b The University of Adelaide, Institute for Photonics and Advanced Sensing, Adelaide, South Australia, Australia; c The University of Western Australia, School of Engineering, Optical+Biomedical Engineering Laboratory, Crawley, Western Australia, Australia; d The University of Western Australia, School of Molecular Sciences, Crawley, Western Australia, Australia; e The University of Western Australia, Medical School, Division of Surgery, Nedlands, Western Australia, Australia; f The University of Adelaide, School of Electrical and Electronic Engineering, Adelaide, South Australia, Australia; g Fiona Stanley Hospital, Breast Centre, Murdoch, Western Australia, Australia; h Royal Perth Hospital, Breast Clinic, Perth, Western Australia, Australia; i University of Surrey, School of Biosciences and Medicine, Surrey Biophotonics, Guildford, United Kingdom; j University of Surrey, Advanced Technology Institute, School of Physics, Surrey Biophotonics, Guildford, United Kingdom; k University of Tasmania, School of Natural Sciences – Chemistry, Hobart, Tasmania, Australia

**Keywords:** optical coherence tomography, fluorescence imaging, fluorescent marker, imaging needle, live cells, breast cancer

## Abstract

**Significance:**

Imaging needles consist of highly miniaturized focusing optics encased within a hypodermic needle. The needles may be inserted tens of millimeters into tissue and have the potential to visualize diseased cells well beyond the penetration depth of optical techniques applied externally. Multimodal imaging needles acquire multiple types of optical signals to differentiate cell types. However, their use has not previously been demonstrated with live cells.

**Aim:**

We demonstrate the ability of a multimodal imaging needle to differentiate cell types through simultaneous optical coherence tomography (OCT) and fluorescence imaging.

**Approach:**

We characterize the performance of a multimodal imaging needle. This is paired with a fluorescent analog of the therapeutic drug, tamoxifen, which enables cell-specific fluorescent labeling of estrogen receptor-positive (ER+) breast cancer cells. We perform simultaneous OCT and fluorescence *in situ* imaging on MCF-7 ER+ breast cancer cells and MDA-MB-231 ER− cells. Images are compared against unlabeled control samples and correlated with standard confocal microscopy images.

**Results:**

We establish the feasibility of imaging live cells with these miniaturized imaging probes by showing clear differentiation between cancerous cells.

**Conclusions:**

Imaging needles have the potential to aid in the detection of specific cancer cells within solid tissue.

## 1 Introduction

Identifying cancer cells within solid tissues, such as breast or prostate, can give critical insights for both diagnosis and treatment. Cancer cells in solid tissue have a wide variety of phenotypes, impacting whether they form well-circumscribed tumors or diffuse masses, the mechanisms driving cell proliferation, and the response to drugs. Identifying cancer cells and monitoring them *in-situ* can offer a means to understand the tumor biology, guide treatment decisions and drug selection, and assess the response to therapy.^1^ Intra-operative identification of cancer cells can also help in the assessment of surgical margins that can have significant implications for reducing the incidence of tumor recurrence.^2^

One method to identify cancer cells is to tag them with a fluorescent label. Fluorescent labels contain a targeting moiety with specific affinity to a molecular target or biomarker of cancer. This targeting moiety is in turn chemically conjugated to a fluorophore that acts as a beacon for the target. Targeted fluorescence imaging is increasingly being explored for intraoperative cancer detection.^3^^–^^6^ However, using fluorescent labels for tumors deep in solid tissue is problematic because optical imaging techniques cannot penetrate deep into tissue. More recently, there has been much attention on near-infrared fluorophores as they may enable greater depths of fluorescence detection.^7^^,^^8^ However, these fluorophores still only enable imaging to a few millimeters deep and often lack target specificity.^4^

Imaging needles provide an alternative approach to enable optical imaging deep in tissue using miniaturized optics that are encased within a hypodermic needle. By inserting the needle into the tissue, they enable optical imaging in the vicinity of the needle tip. Imaging needles have been explored in solid tissue to identify brain blood vessels at risk of hemorrhage in humans,^9^ brain tumors in a mouse model,^10^ biopsy sites in sheep lung,^11^ breast tumor margins^12^^,^^13^ and prostate tumors *in vivo*.^14^ Most imaging needles have utilized optical coherence tomography (OCT), which provides information on the microarchitecture of the tissue, but lacks the biochemical information available through targeted fluorescence.

In breast cancer, OCT has some ability to identify cancerous tissue, particularly its morphological architecture, by its high optical scattering. However, other non-cancerous tissues, such as fibrous regions rich in collagen, have been shown to exhibit similarly high optical scattering.^15^ Conversely, fluorescent cellular marking of malignant breast tissue has been demonstrated through pre-operative administration of 5-aminolevulinic acid hydrochloride, but non-malignant proliferative cellular changes in tissue, including hyperplasia, columnar cell change, and atypical ductal hyperplasia, were also shown to give rise to a false-positive fluorescence signal.^16^ The combination of multiple modalities provides a potential mitigation to the limitations of each individual imaging technique. In particular, the combination of OCT + fluorescence offers the OCT imaging resolution for visualizing microarchitectural changes associated with many forms of malignancy,^17^ detection of critical structures such as blood vessels,^9^ and highly specific fluorescence detection of certain forms of cancer.^16^

Miniaturized fiber-optic probes that can augment OCT with fluorescence to perform multimodal imaging have been demonstrated in both intravascular^18^^–^^20^ and endoscopic^21^ applications. Recent work established the feasibility of inserting a multimodal fiber-optic probe through a guide needle, using both imaging modalities to improve yield in lung biopsies.^22^ Previous work by our team proposed a side-facing OCT + fluorescence imaging needle and demonstrated cell-specific imaging in fixed, non-cancerous tissue using fluorescently labeled antibodies.^23^

In this paper, we extend this work to the imaging of live cancer cells. This brings multiple challenges as many fluorescently labeled antibodies are not appropriate for live cell imaging. To address this, we developed a fluorescent analog of the drug tamoxifen with specific binding affinity for estrogen receptor-positive (ER+) breast cancer cells, achieved using a BODIPY^®^FL fluorophore conjugate and a hydrophilic linker.^24^ In parallel, we developed a multimodal OCT + fluorescence imaging system and interfaced this with a multimodal imaging needle, based on a design utilizing double-clad fiber (DCF) and all-fiber lensing elements, to allow for simultaneous and co-localized detection of the OCT and fluorescence emission light. We demonstrate live imaging of breast cancer cells with an OCT + fluorescence imaging needle with an outer diameter of 570  μm. The OCT images show architectural differences between cell types, while the fluorescence signal provides co-located cell-specific labeling to differentiate ER+ and estrogen receptor-negative (ER−) cancer cell types. To the best of our knowledge, this is the first demonstration of the use of an OCT + fluorescence imaging needle with specifically labeled live cancer cells. This study establishes that this highly miniaturized optical design has sufficient sensitivity to detect fluorescence from live cells.

## 2 Materials and Methods

### 2.1 Dual-Modality Imaging System

**Fig. 1:**
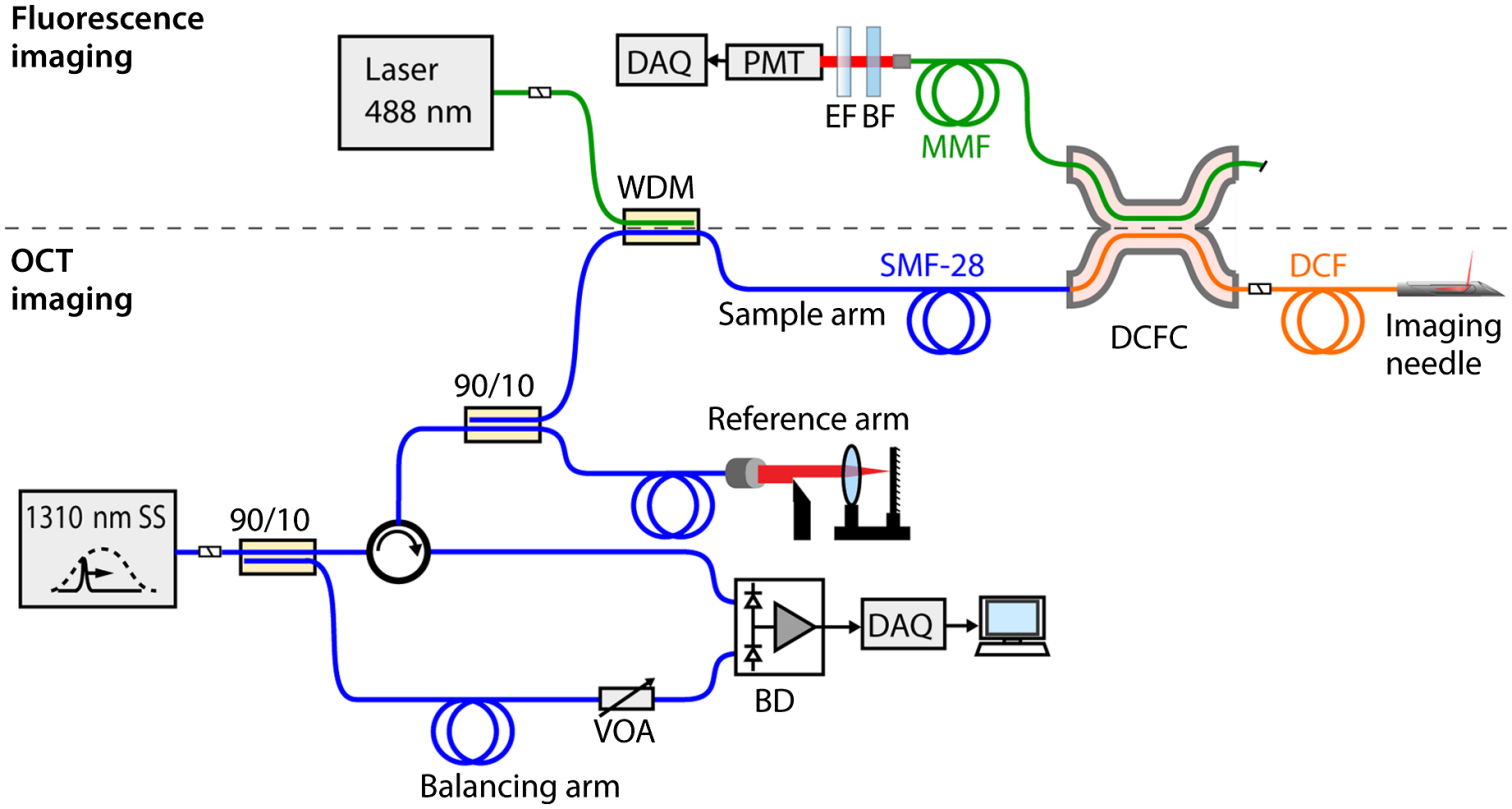
Schematic of the dual-modality OCT + fluorescence imaging system. SS, swept source; WDM, wavelength-division multiplexing coupler; SMF-28, single-mode fiber; MMF, multimode fiber; DCF, double-clad fiber; DCFC, double-clad fiber coupler; EF, long-pass emission filter; BF, blocking filter; PMT, photomultiplier tube; VOA, variable optical attenuator; BD, balanced photodetector; DAQ, data acquisition card.

The dual-modality imaging system has been previously described and characterized.^23^ A schematic of the system is shown in Fig. 1. Briefly, the OCT imaging hardware consists of a wavelength-swept laser source with a center wavelength of 1310 nm, bandwidth of 100 nm, sweep rate of 50 kHz, and output power of 40 mW (Axsun Technologies Inc.). The source light is directed through single-mode fiber (SMF28, Corning) to a circulator and then to a 90/10 coupler that sends the light into the sample (90%) and reference (10%) arms for imaging. Returning light is directed back through the coupler and circulator for detection with a dual-balanced photodetector (PDB420C-AC, Thorlabs). This system implements a manual-balancing variant of balanced detection, as described in Ref. 25. Unlike some other approaches to balanced detection, this scheme may be utilized for both dual-arm and common-path OCT probes. An additional 90/10 coupler at the source is used to tap off a small proportion of the source light, which is directed through a path-length matched balancing arm of the system. A variable optical attenuator (VOA50-APC, Thorlabs) in the balancing arm is used to scale the source light to be comparable to the interference signal. The scaled source signal is then subtracted from the interference signal at the balanced detector to suppress the intensity noise of the source. Acquisition of the detected signal is accomplished using a digitizer card (ATS9350, AlazarTech, Canada). A Hann window was used for spectral shaping, providing a good compromise between maintaining axial resolution while minimizing sidelobes in the axial point spread function. This is particularly relevant to the work presented here as the sidelobes of the strongly reflecting interfaces of the microscope coverslips used to protect the cell samples may otherwise corrupt the *en face* image of the adjacent cell layer. The complete custom-built imaging system with spectral shaping gives an axial resolution in air of 13  μm, corresponding to ∼9  μm in tissue (refractive index, n=1.4). With the imaging needle as the sample arm, the detection sensitivity was measured to be 100 dB, which is comparable to a typical OCT benchtop system.

The fluorescence imaging hardware consists of a 488-nm frequency-doubled semiconductor laser for fluorescence excitation (Sapphire SF 488, Coherent Inc.). The laser provides an output power that can be adjusted between 90  μW and 8 mW at the sample. The light from the laser is injected through a wavelength-division multiplexing coupler (INT-MSI-1300, Thorlabs) into the core of the SMF-28 fiber of the sample arm. The SMF-28 fiber is then connectorized to a length of DCF (SM-9/105/125- 20A, Nufern) that forms the input arm to a custom-built double-clad fiber coupler (DCFC) (DC1300LE, Castor Optics, Canada). The coupler is a 4-port optical component comprising a length of DCF and a length of multimode fiber (MMF) that are fused at a point along their length using a custom fusion-tapering process, detailed previously.^26^ The DCF delivers both the OCT illumination and fluorescence excitation through its 9-μm core to the sample. The 105-μm-diameter inner cladding of the DCF then provides a high-numerical aperture (NA) optical path through which emitted sample fluorescence can be collected. For the fluorescence signal, injecting the excitation light through the fiber core but detecting fluorescence emission through the inner cladding results in a different NA for each light path. The final NAs of the illumination/excitation and collection/emission paths are limited by the imaging optics of the needle. Sample fluorescence collected in the inner cladding passes to the DCFC and then is detected using a photo-multiplier tube (PMT) (9136B, Electron Tubes, United Kingdom). A 488-nm blocking filter (NF02-488S-25, Semrock) and a 500-nm long-pass emission filter (FF01-500/LP-25, Semrock) minimize the detection of source and background noise. Fluorescence data were acquired simultaneously with OCT data using a separate digitizer card (NI PCIe-6351, National Instruments Corporation). Referring to Fig. 1, 59% of the 488-nm fluorescence excitation light coupled into the wavelength-division multiplexing coupler (WDM) and 69% of the 1300-nm OCT light coupled into the WDM are transmitted to the DCF fiber output of the DCFC. For fluorescence detection, 85% of the light collected in the DCF inner cladding is coupled to the MMF output of the DCFC.

The achievable fluorescence detection sensitivity of the system was measured by imaging glass tubes filled with fluorescein solutions of variable concentration, diluted in pH 9.5 buffer, and with the fluorescence excitation power set to 90  μW. The minimum detectable concentration of fluorescein was 1.5 nM. Increasing the source power did not increase the detection sensitivity as the noise floor from autofluorescence in the imaging system also increased. It is worth noting that not all forms of noise increase as a function of source power, but we found autofluorescence to be the limiting factor in our experiments. Phototoxicity is largely driven by damage to cell membranes or their components and damage to DNA.^27^ Minimizing irradiance by reducing excitation light power is an effective strategy for reducing phototoxicity in live cell imaging.^28^ Therefore, the lowest selectable power of 90  μW was used in live cell imaging to minimize the risk of damage to cells. It is worth noting that our multimodal probe was designed to have a lower NA than is common in fluorescence-only systems to optimize the OCT imaging depth. Although this results in lower spatial resolution (illumination spot size), it also decreases the irradiance at the focus, further limiting potential cell damage.

### 2.2 Imaging Needle

#### 2.2.1 Imaging needle design

**Fig. 2:**
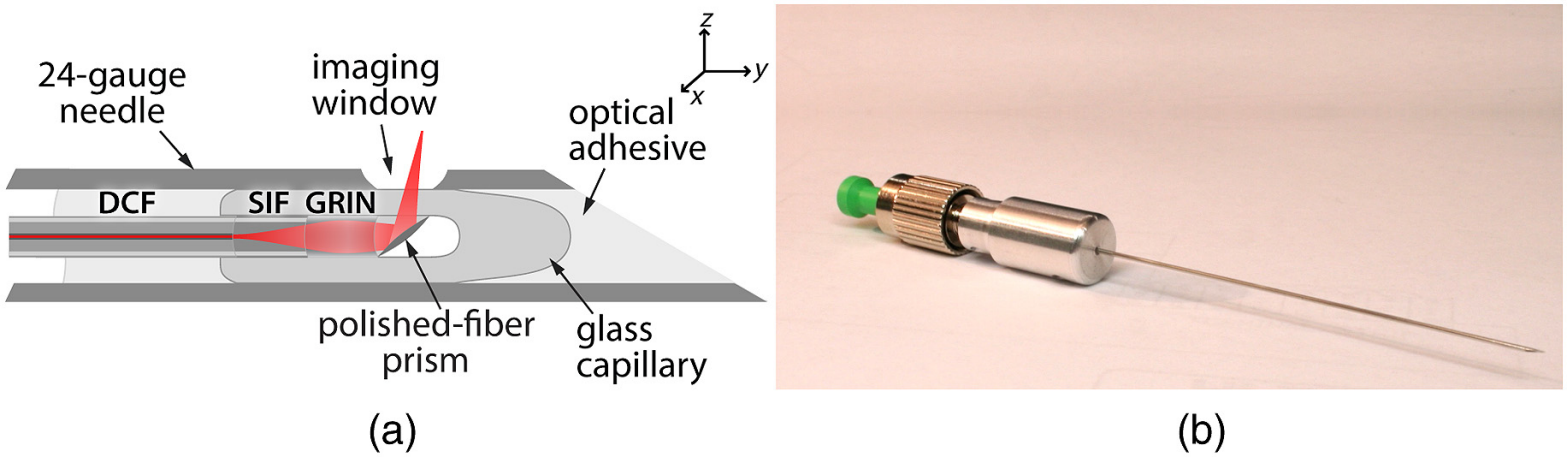
(a) Schematic of the dual-modality imaging needle. (b) Photo of the manufactured imaging needle. DCF, double-clad fiber; SIF, step-index multimode fiber; GRIN, gradient-index multimode fiber.

We previously demonstrated an imaging needle that could simultaneously achieve high OCT imaging and fluorescence detection sensitivities.^23^ The needle utilizes a fused-fiber design with high-quality optical interfaces and very low loss as shown in Fig. 2. The lensing optics consist of fiber elements sequentially fused to the end of a DCF pigtail, specifically, a large-core step-index fiber that allows the beam to expand from the DCF core and a graded-index (GRIN) fiber that focuses the beam to a point in front of the needle. A third section of pure silica fiber is fused onto the GRIN fiber and polished to an angle of 48 deg to form a prism that redirects the light almost perpendicularly to the needle using total internal reflection (TIR). The lensing fibers are fully enclosed in a glass capillary, which is subsequently collapsed and tapered to introduce an air gap at the polished fiber prism to ensure a TIR interface. The entire assembly is glued into a 24-gauge (570  μm) hollow needle with an electrochemically etched side-viewing window for the light to exit. The optical glue is kept clear of the imaging window to maintain beam integrity and minimize loss and background fluorescence.

The characteristics of the output beam, including the focal spot size, the working distance, and the Rayleigh range, can be tuned by careful selection of the lengths of each fused fiber section. This is true for both OCT illumination and fluorescence excitation, which exhibit similar characteristics since their foci are axially collocated (assuming that chromatic aberration of the fiber lenses is negligible). We used in-house optical modeling software with paraxial ray-matrix transformations for Gaussian beams to simulate the output beam for various lengths of GRIN and step-index fiber. Details of the analysis methodology along with a more complete analysis of the focusing optics for these fiber probes have been presented previously.^29^ In designing an imaging needle for visualizing live cells, we chose fiber lengths that would yield an output beam with a spot size of around 15  μm and a mid-range illumination NA of 0.07. This choice strikes a compromise between OCT depth range, which is maximized by low illumination NA, and fluorescence collection efficiency, which requires high collection NA. Illumination and collection NA are coupled as the latter is determined by the effective fluorescence collection aperture of the probe (approximated as the GRIN fiber core area) and the working distance, which becomes shorter when the illumination NA is increased. We found that the lengths required to achieve the desired output beam were 430  μm for the step-index fiber and 180  μm for the GRIN fiber. Our final needle was manufactured with fiber lengths close to the required lengths and gave a predicted output beam (in water) with a working distance of 400  μm and a slightly astigmatic focal spot (due to the curved surface of the exit window) with a full-width at half-maximum (FWHM) spot-size of 12  μm in the x direction and 16  μm in the y direction (assuming n=1.33).

It is noteworthy that the properties of the light beam are different in air and water due to the difference in the refractive index. Modeling the light beam in water provides a better indicator of the beam properties in deep tissue imaging because of the refractive index of tissue is closer to that of water than air. However, it was impractical to immerse the beam profiling setup in water. For this reason, when characterizing our fabricated imaging needles, we measured the beam profile in air to validate our model and then used this model to simulate the corrected beam in fluid.

One can obtain an approximation of the fluorescence collection NA of this probe by calculating the marginal ray angle $\Theta_{\max}$ of returning fluorescence light emanating from a point at the working distance of 400  μm, which is captured by the 100-μm diameter core of the GRIN fiber. Taking into account the total path length in water and glass up to the GRIN fiber end face, we obtain an approximate fluorescence collection NA of 0.12 (where $\mathrm{NA}=n_{w}\cdot \;\sin(\Theta_{\max})$ and $n_{w}=1.33$ is the refractive index of water).

#### 2.2.2 Imaging beam characteristics

To validate the output beam of our manufactured imaging needle and determine whether it achieved the desired optical characteristics, as predicted from our modeling simulation, we characterized the beam produced for both OCT and fluorescence wavelength ranges. The OCT and fluorescence beams are characterized in different ways because of the differences in their imaging mechanisms. OCT is based on an optical backscatter, which does not experience a change in wavelength. Hence, the characterization of the OCT imaging optics is achieved by measuring the beam emitted from the imaging needle. In fluorescence imaging, excitation and emission occur at different wavelengths. To account for this, characterization of the fluorescence imaging optics is achieved by collection of emission from a well-characterized fluorescent object that has been excited by light emitted from the imaging needle.

For OCT beam characterization, we used a CCD beam profiler positioned in front of the imaging window, with light from a 1300-nm superluminescent diode injected into the DCF core providing the source. Light exiting the needle was directed to the center of the CCD camera, and two-dimensional intensity maps of the resulting beam were recorded at discrete distances from the imaging window, in 20-μm increments from 0 to 1.2 mm. The FWHM beam diameters were calculated by taking the 2D intensity maps recorded at each depth and performing a Gaussian fit to profiles extracted from a line through the center of the beam in both the x and y axes. From these measured profiles, the expected working distance, spot size, and OCT resolution of our imaging needle in tissue were determined.

We next characterized the fluorescence imaging properties of the needle by estimating its fluorescence point-spread function (PSF). To do this, we used a small fluorescent bead as a point source placed in front of the imaging window at different depths. From the recorded images, the PSF at each location was estimated. We used 7.7-μm fluorescent beads suspended in water (AlexaFluor^®^ 488 Reference Standard 886, Bangs Laboratories Inc.), which we first dehydrated on a glass coverslip and then covered with a semi-cured silicone matrix. After fully curing the matrix, the beads remained firmly embedded in the surface of the matrix and were ready for imaging under water immersion. For imaging, we used a two-axis raster scanning stage (H2W Technologies, Santa Clarita, California) operated in closed loop, with the trigger signals for both OCT and fluorescence being derived from the optical encoder pulses to provide distortion-free and equidistant sampling of the image. The side-viewing needle was positioned as close as possible above and parallel to the matrix, with a drop of water between the needle and the matrix to avoid the beam distortions that would be caused by an air interface. A single bright fluorescent bead was identified by translating the matrix under the needle while viewing the fluorescence signal. Fluorescence images of the bead were then captured by xy raster scanning of the stage with data captured in 4  μm increments in both the x and y axes. Images of the bead were recorded at increasing distances from the needle window, from 330  μm up to 1.2 mm. From the recorded 2D fluorescence intensity maps of the bead, cross sectional profiles of the PSF in both the x and y axes were obtained by averaging 10 linescans about the center of the bead in each respective axis. A Gaussian curve was then fitted to each cross section to estimate the FWHM diameter of the PSF as a function of distance from the needle.

### 2.3 Live Cell Labeling and Imaging

#### 2.3.1 Fluorescent label synthesis

To achieve cell-specific fluorescence labeling of live cells, we synthesized a fluorescent analog of the selective estrogen modulator drug tamoxifen. Tamoxifen binds to and modifies the estrogen receptors (ERs) in cells. ERs play an important role in regulating processes, such as cell proliferation and differentiation,^30^ with the result that over-expression of ERs is present in ∼70% of all breast cancers.^31^ Therefore, tamoxifen is used extensively to treat ER+ breast cancers by blocking their growth.

**Fig. 3:**
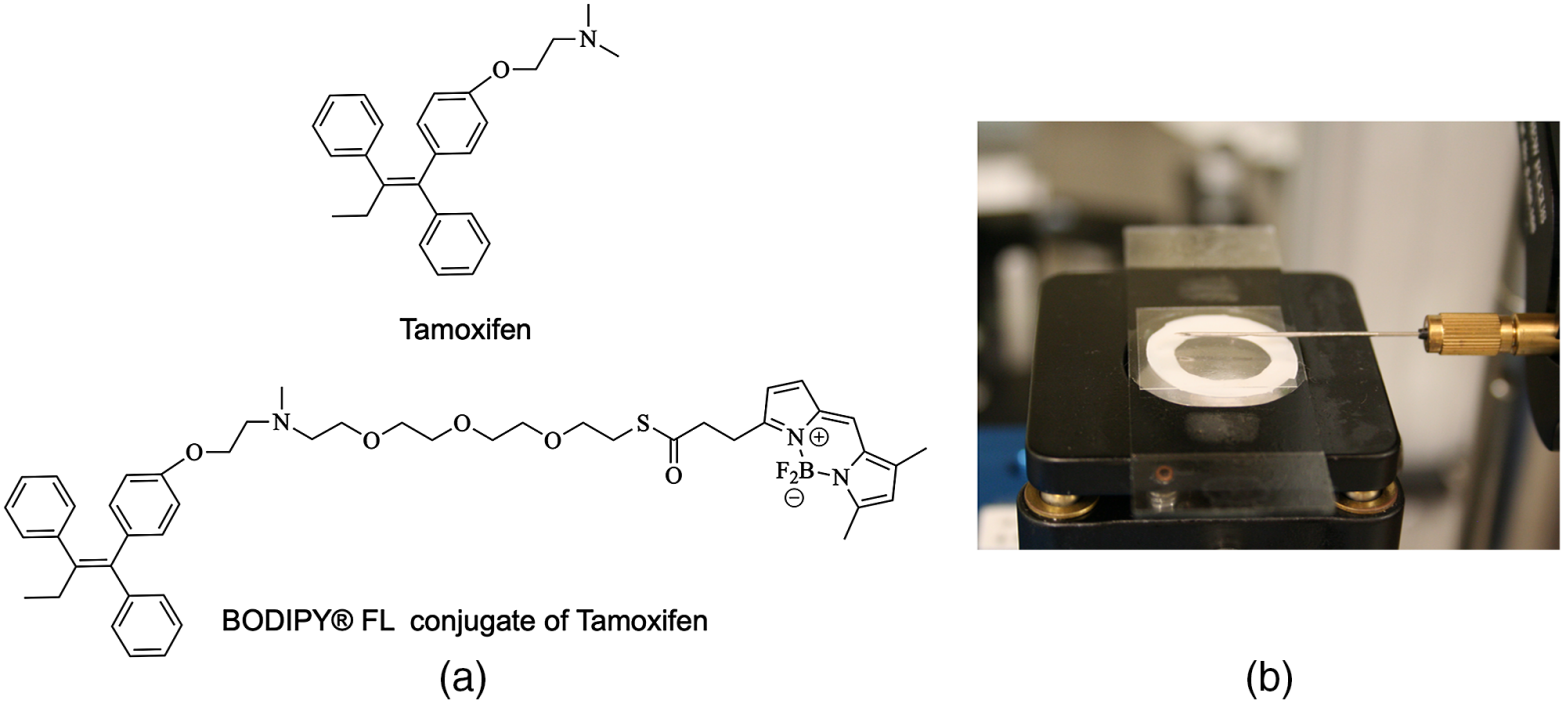
(a) The chemical structure of the drug tamoxifen and its synthesized analog with BODIPY^®^ FL conjugate. (b) Experimental setup for live cell imaging.

In earlier work, we have reported details of the synthesis of our fluorescent analog of tamoxifen labeled with a BODIPY^®^ FL fluorophore.^24^^,^^32^ The chemical structure of tamoxifen and our synthesized analog are presented in Fig. 3. Excitation of the fluorescent analog at 488 nm results in a fluorescence emission peak above 500 nm. We chose to use BODIPY^®^ FL as it has previously been shown to be cell permeable, allowing for more extensive binding to receptors both on the cell membrane and internally within the cell.^33^

#### 2.3.2 Cell culture and imaging

We prepared cultures of two breast cancer cell lines for incubation with the synthesized fluorescent label: ER+ Michigan Cancer Foundation-7 (MCF-7) cells and ER− M.D. Anderson - Metastatic Breast 231 (MDA-MB-231) cells, with the goal of distinguishing the two cell lines through specific fluorescent labeling. For incubation and imaging, cells from each cell line were cultured onto glass coverslips in sterile six-well plates at a concentration of $4\times 10^{5}\text{ cells}/\mathrm{ml}$. Two wells of each culture were incubated for one hour with the fluorescent tamoxifen analog diluted to a concentration of 10  μg/ml in culture media. The cells were then rinsed three times in phosphate-buffered saline to remove unbound tamoxifen and immersed in fresh cell culture media (without fluorescent tamoxifen). Control samples were prepared in two additional wells without the fluorescent tamoxifen. Well-plates were prepared in duplicate with the first plate used for initial confocal imaging to verify uptake of the fluorescent drug before needle imaging and the second plate used for needle imaging.

Imaging with the side-viewing needle was performed by positioning the needle 250  μm above and parallel to the coverslip to be imaged, with the cultured cells attached on the underside of the coverslip and a drop of water placed between the needle and the coverslip to eliminate air from the optical path. A silicone ring (0.5-mm thickness) around the underside of the coverslip allowed the cells to be kept immersed in culture medium, ensuring that they remained alive during imaging. To obtain OCT and fluorescence images, the mounted coverslip was secured on a two-axis raster scanning stage and translated at a constant speed over a distance of 5×5  mm with 5-μm spacing between data captures. Scanning was not optimized for speed. However, in parallel work, we have explored alternate scanning mechanisms for OCT + fluorescence fiber probes that are appropriate for applications such as intravascular imaging.^34^ All imaging was performed in the dark and at room temperature. Immediately after imaging, the cell cultures were fixed in formalin and subsequently imaged with confocal microscopy for verification of fluorophore labeling.

OCT and fluorescence data were processed and reconstructed using software implemented in-house in the C++ programming language. 2D OCT images corresponding to an *en face* plane located within the cell layer were selected for display from each 3D OCT dataset. Fluorescence data were displayed as a projection of detected fluorophores at each xy point and were inherently co-registered with the OCT image data by virtue of simultaneous acquisition through the dual-modality imaging needle.

## 3 Results

### 3.1 Imaging Beam Characterization

**Fig. 4:**
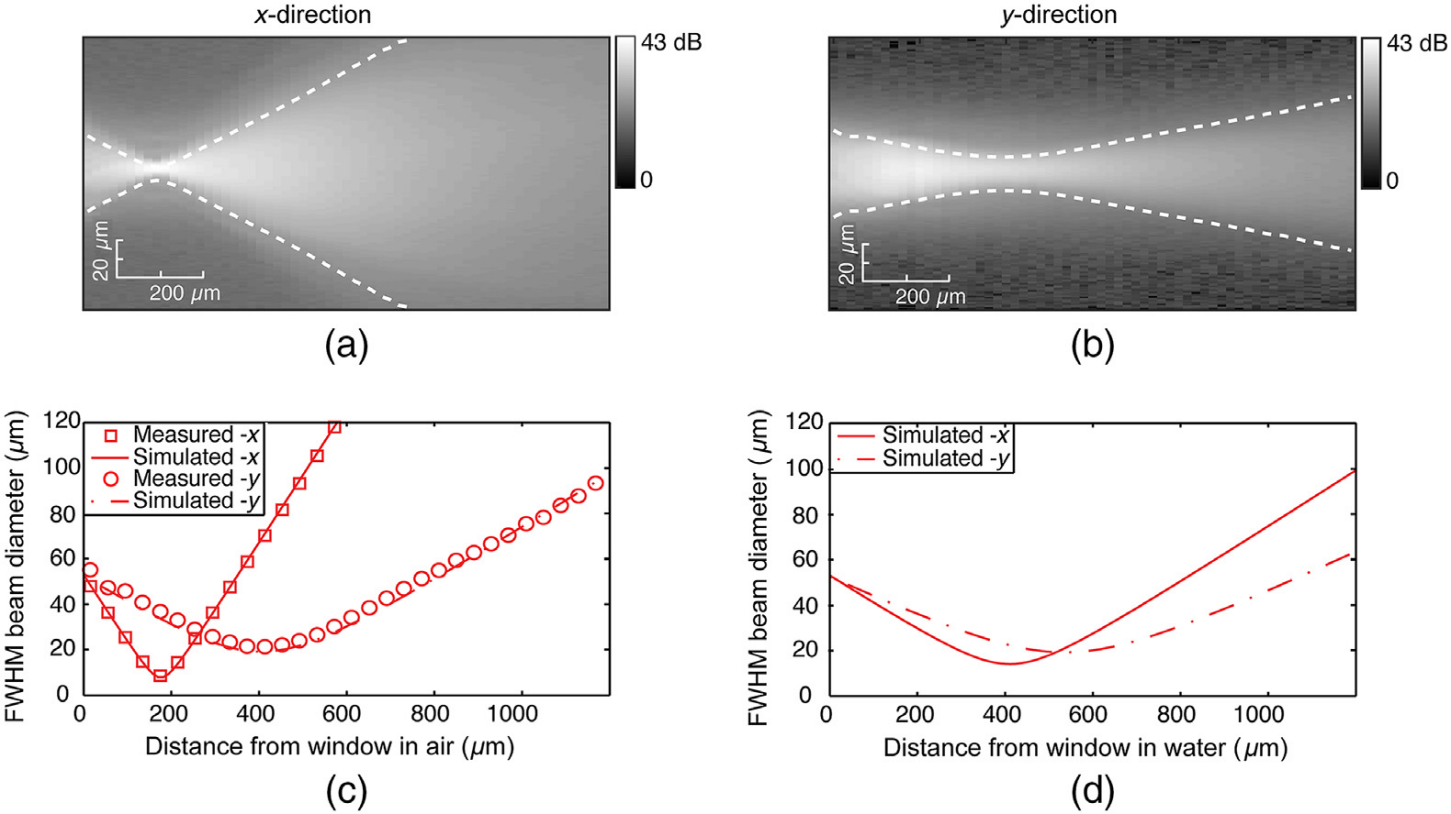
(a) and (b) Recorded OCT beam profiles in air showing the extracted intensity maps for the xz and yz planes, respectively. (Measured FWHM beam diameters are shown as dashed white lines.) (c) Comparison plot of the measured FWHM beam diameters with simulated values. (d) Plot of the simulated FWHM beam diameters in water.

Figures 4(a) and 4(b) show a map of the recorded beam intensities extracted over the xz and yz planes, respectively. The measured FWHM beam diameters at each depth are overlayed as dashed white lines.

We compared measured FWHM values in air with those obtained from our simulation, with the comparison shown in Fig. 4(c). We see that there is very good agreement between our simulated output beam parameters in air and the actual measured output beam. Correcting the beam for water instead of air, we were then able to predict the expected output beam in water (n=1.33). The results of the simulated output beam in water are shown in Fig. 4(d). From this simulation, we calculated that the imaging needle achieves a working distance in water of 410  μm and an astigmatic FWHM spot size of 11.9 and 16.3  μm in the x and y directions, respectively.

**Fig. 5:**
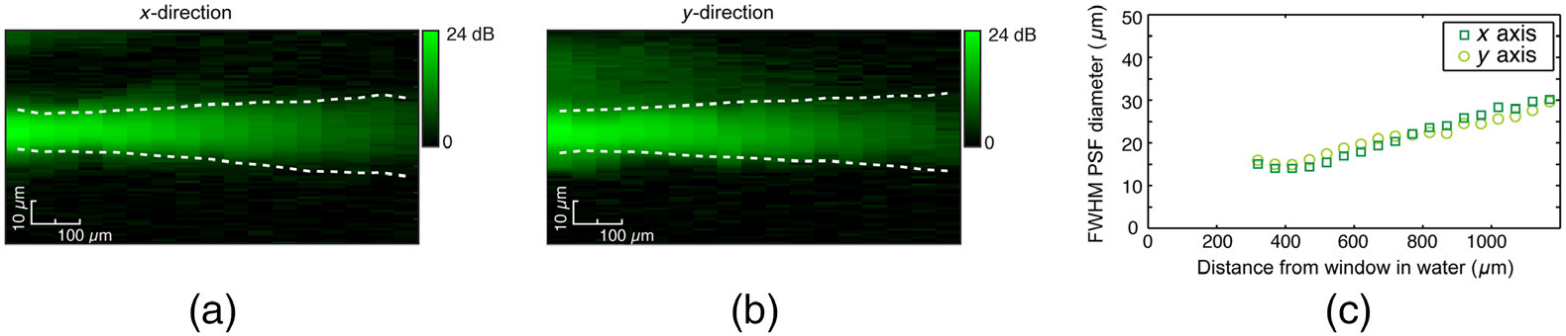
(a) and (b) Recorded fluorescence PSF data for a 7.7-μm fluorescent bead versus distance from the needle window in the xz and yz planes, respectively. (Extracted FWHM PSF diameters are shown as dashed white lines.) (c) Plot of the extracted FWHM diameters of the PSF showing the change in resolution as a function of depth.

Measured fluorescence profiles extracted from images of a fluorescent bead at increasing distances from the needle are shown in Fig. 5. The resulting FWHM diameters are shown by the dashed white lines in Figs. 5(a) and 5(b) and are plotted in Fig. 5(c). It is useful to observe that the PSF is narrowest at ∼400  μm from the needle window, where it reaches an FWHM diameter of 12  μm in the x direction and 13  μm in the y direction. The beam width is doubled at 1.2 mm from the needle window. Therefore, the best fluorescence resolution is reached for a fluorophore at 400  μm. However, this must be balanced against the decrease in fluorescence detection sensitivity for increasing distances from the needle. The fluorescence efficiency was found to drop off rapidly at a rate of −23  dB/mm, suggesting that fluorescence detection is maximized for a fluorophore closest to the needle window. We note that this is expected to be more significant in solid breast tissue, which is highly optically scattering in the range from 500 to 600 nm,^35^ resulting in more rapid reduction in fluorescence sensitivity with distance.

### 3.2 Live Cell Imaging Results

**Fig. 6:**
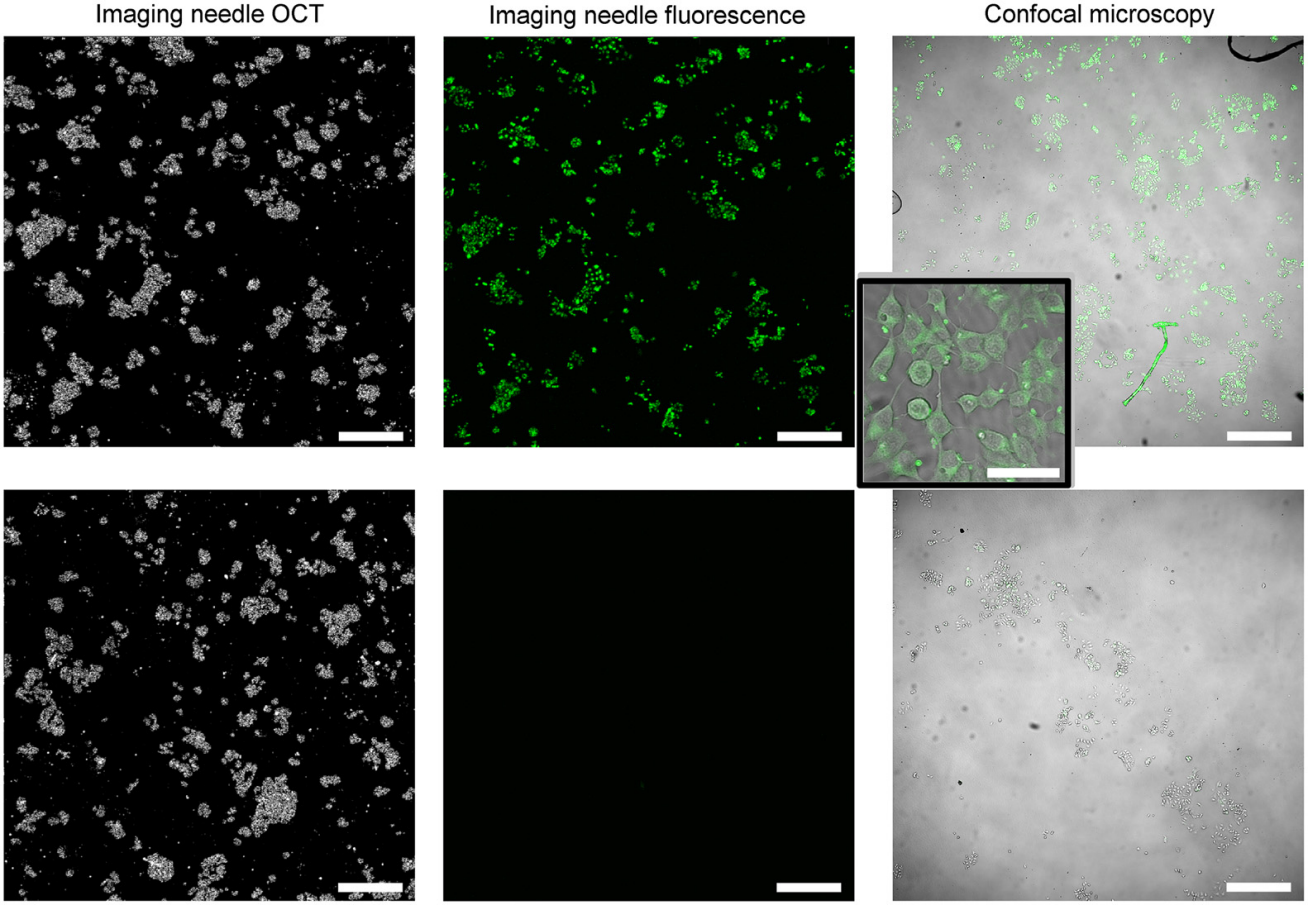
Dual-modality needle imaging results of live MCF7 (ER+) breast cancer cells. Top row: cells incubated with a fluorescent tamoxifen analog showing cell-specific fluorescence. Bottom row: control sample of cells with no fluorescent label showing no autofluorescence. Left: needle OCT of live cells. Center: needle fluorescence of live cells. Right: confocal microscopy images of sample acquired after fixation at ×4 magnification. It is noteworthy that the confocal image is not co-located with the needle images. It is also noteworthy that the unlabeled cells show no measurable autofluorescence; hence the center image bottom row appears black. Scale bars: 500  μm. Inset: zoomed confocal microscopy image showing cell uptake of fluorescent tamoxifen analog. Inset acquired after fixation at ×20 magnification. Scale bar in inset image: 50  μm.

**Fig. 7:**
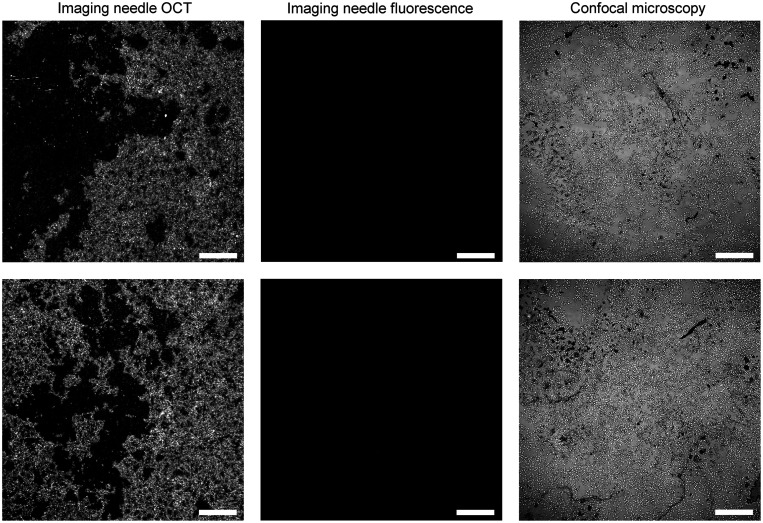
Dual-modality needle imaging results of live MD231 (ER−) breast cancer cells. Top row: cells incubated with a fluorescent tamoxifen analog showing no specific fluorescence labeling as expected. Bottom row: control sample of cells with no fluorescent label showing no autofluorescence. Left: needle OCT of live cells. Center: needle fluorescence of live cells. Both labeled and unlabeled cells (top and bottom rows) show no measurable fluorescence; hence the images appear black using the same intensity scale as Fig. 6. Right: confocal microscopy images of sample after fixation. Acquired at ×4 magnification. Scale bars: 500  μm.

The results of imaging live cell cultures with our imaging needle are shown in Figs. 6 and 7 for ER+ and ER− cells, respectively. Simultaneous needle OCT *en face* images and needle fluorescence images are presented in the two panels on the left for the case of incubation with the fluorescent tamoxifen label (above) and for the control without the fluorescent label (below). The confocal image of the sample obtained after fixation is presented in the right panel of each figure. It is noteworthy that the confocal images acquired after fixation are not co-located with the live needle images and hence show a different representative region of the cells.

In Fig. 6, the OCT image depicts clusters of high optical backscattering, with a distribution that displays the structure of the cell culture. From the OCT image, we see that both control and labeled cells were successfully cultured. The fluorescence image (top row, center) shows well-circumscribed points of high fluorescence, which appear in the same location as the high-backscattering clusters in the OCT image. The images are inherently collocated, which suggests that the observed fluorescence is coming from the cells. Confirmation that the cultured cells are fluorescent is established by the confocal image on the right, acquired at ×4 magnification. These images show clear demarcation of the fluorescent cell boundaries. The confocal inset, acquired at a magnification of ×20, also shows that the detected fluorescence is internal to the cells, which is consistent with the expected localization of the BODIPY^®^ FL conjugate. Artifacts, observable as hair-like structures in the confocal image, are due to debris introduced during fixation of the cells. Comparing with the control images in the lower panels in Fig. 6, we confirm that the fluorescence is due to the incubated fluorescent label and not the cell autofluorescence because there is no fluorescence observed in the control cells. This is again confirmed in the confocal image. We conclude that there was successful labeling of the cells with the fluorescent tamoxifen analog, and this is clearly detectable with our OCT + fluorescence imaging needle.

Figure 7 shows a high specificity of labeling with our fluorescent tamoxifen analog as ER− cells do not exhibit fluorescence. In the upper panel, the OCT image depicts a successful cell culture consistent with the growth of MDA-MB-231 (ER−) cells, which are much denser than MCF7 (ER+) cells. This growth pattern is validated in the confocal image acquired after fixation of the sample. However, ER− cells incubated with the fluorescent label show no fluorescence detected in the needle image. The lack of fluorescence is validated by the confocal image on the right. This suggests that the fluorescent tamoxifen analog is not taken up by the ER− cells. Therefore, we have confirmed that uptake of the label is cell-specific and that our imaging needle is both sensitive and specific enough in detecting labeled ER+ breast cancer cells.

**Fig. 8:**
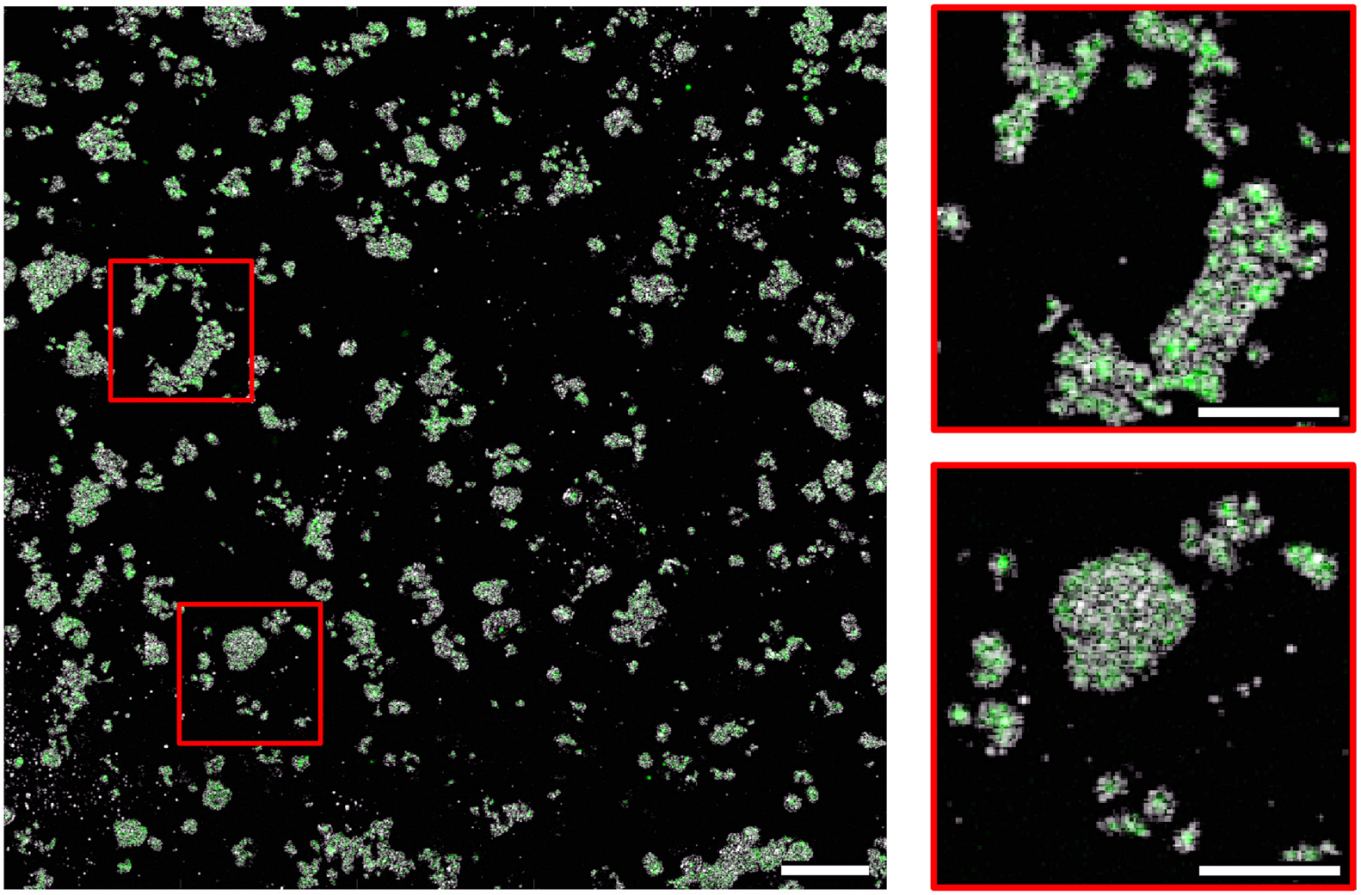
Left: combined OCT+fluorescence dual-modality needle image of live MCF7 (ER+) breast cancer cells. Scale bars: 500  μm. Right: zoomed images of regions delineated with a red box. Scale bar in zoomed images: 250  μm. The zoomed images depict the clear overlap of the complementary signals, highlighting the value of this imaging needle for simultaneous and co-registered dual-modality imaging.

Finally, in Fig. 8, we demonstrate the powerful nature of the imaging needle for dual-modality imaging. The unique design of the needle optics means that both OCT and fluorescence images are inherently co-registered through the same focusing optics. Overlaying the OCT and fluorescence needle images, we, therefore, gain additional insight into the combined microstructure and biochemical composition of the sample. This is demonstrated in the zoomed images in Fig. 8. In the dual-modality image, clear clusters of cells are made visible by the simple superposition of the OCT and fluorescence data. The fluorescence image pinpoints the specific cell type, while the extent of cells is evident from the OCT image. With the aid of the OCT image, it becomes possible to differentiate cells from artifacts in the fluorescence image. With the fluorescence image, it becomes possible to differentiate cells from debris in the OCT image.

## 4 Discussion

We have demonstrated an imaging needle and system capable of visualizing live cells tagged with a cell-specific fluorescent label. We have chosen to demonstrate this in an *in-situ* scenario to allow for validation against confocal microscopy. However, earlier work with ex vivo tissue labeled with fluorescent antibodies has shown that simultaneous OCT and fluorescence imaging is feasible by inserting an imaging needle into solid tissue.^23^ Our imaging needle uses dual-modality imaging to identify cells by combining sensitive fluorescence detection with simultaneous, co-registered OCT. Although the resolution of OCT is typically insufficient to differentiate individual cancer cells, it can usefully augment fluorescence detection by providing microstructural information that contextualizes the fluorescence or by providing measurements of complimentary tissue properties that change with malignancy, such as optical scattering.^15^ We found that the imaging needle was highly sensitive and was able to detect a synthesized fluorescent analog of the drug tamoxifen specifically bound to live ER+ breast cancer cells. This analog has the potential for labeling breast cancer cells in solid tissue, with significant intra-operative implications. One possible application could be the detection of residual ER+ breast cancer cells at the margins of excision during breast-conserving surgery. The imaging needle also represents a critical step in bridging the gap between available selective fluorescent drugs and the exploration of their use *in vivo*. Imaging needles provide a pathway to research drugs developed for solid tissue diseases, without the need for invasive removal of tissue, therefore, offering the possibility of true *in vivo* analysis and longitudinal studies.

In applying our imaging needle for *in situ* detection of fluorescently labeled cells, there were several competing considerations. First, the fluorescence sensitivity needed to be high enough to detect the low-level emission from fluorophores with the potential to be internalized into live cells, which we found to be significantly lower than in our earlier work using fluorescently labeled anti-bodies bound to the external membrane of fixed cells.^23^ The achievable sensitivity of the system is limited by the autofluorescence of the tissue and the background level of fluorescence generated by the interaction of excitation and emission light with the imaging system, which has not previously been well characterized. This background is dependent on the excitation and emission wavelengths of the fluorophore. We found that, for our system, background fluorescence was drastically reduced by removing plastic fiber jackets and materials in the beam path that could be excited to emit fluorescence at similar wavelengths to our fluorescent label (500 nm and above).

The use of DCF also gives rise to OCT imaging artifacts that are not present in systems utilizing standard single-mode fibers (e.g., SMF-28). Because there are dual paths through which light can travel in DCF (through the core and through the inner cladding), it is possible for light to leak from one path to the other and propagate so as to create imaging artifacts. We observed this occurring in our system at the fiber connection where the DCF fiber from the coupler meets the DCF fiber in the sample arm, providing an opportunity for light to leak into the inner cladding. Once light has leaked into the inner cladding, it propagates as a faster multimodal signal due to the lower refractive index of the cladding. It is then focused onto the sample by the needle optics and backscattered and collected into the DCF core. This contributes an additional interference signal that reaches the detector more rapidly than the signal produced by light propagating entirely within the DCF core. If the optical pathlength difference between the two signals is within the OCT imaging range, the additional signal will be visible as a ghost image of the sample. We observed that this phenomenon was exacerbated by bends in the fiber, which may mix light between modes and between the two paths. However, if the sample arm fiber is sufficiently long, the optical path difference between the signals will be great enough that the ghost image is moved out of the imaging range. Recent work by Tanskanen et al.^36^ characterized these artifacts as higher-order modes propagated through the DCF inner cladding but with their energy primarily in the core and showed that the offset of the ghost image increased linearly with DCF fiber length. For our system, we found that a sample arm DCF fiber length of at least 2 m was sufficient to move the artifact out of the imaging range.

The value of dual-modality imaging within a needle is apparent in this study. However, we note that the fluorescence image is obtained as a projection, with fluorescence aggregated from over several hundred microns in depth. This is because of limitations in our ability to fabricate high NA micro-lenses at this scale, as well as the need to maintain a low NA to maximize the imaging depth of the OCT signal. However, the high-resolution axial sectioning ability of OCT provides useful depth information that can provide insight into the tissue structures giving rise to the fluorescence. In this way, OCT aids interpretation of the origins of the fluorescence signal.

The measurement of quantitative tissue fluorescence may, in the future, be possible with our dual-modality needle. Quantifying fluorescence requires correction for signal loss that occurs due to absorption and scattering of the excitation and emission light. Previous researchers have proposed methods to quantify fluorescence in tissue, typically through the use of a correction factor.^37^^,^^38^ For example, white light diffuse reflectance has been used to infer tissue optical scattering and absorption at fluorescence wavelengths, which is then used to correct for attenuation of the fluorescence signal.^18^ The use of dual-modality OCT and fluorescence imaging needles may offer an alternative approach using OCT to estimate the correction factor required. Other work in tissue identification and segmentation gives rise to the possibility that the fluorescence signal may be corrected for tissue-specific values of scattering and absorption by analysis of the OCT image.^15^ This is analogous to techniques used in other dual-modality clinical imaging systems, such as PET-CT, where the PET scan is attenuation corrected using co-registered X-ray CT data.

## 5 Conclusion

This study represents a useful step toward detecting diseased cells in solid tissue through the pairing of a dual-modality imaging needle with a cell-specific fluorescent label. The imaging needle has great potential for use in applications ranging from the detection of residual cancer during surgical excision of a tumor to the assessment of cell function within tissue in response to drugs. Our imaging needle represents a significant advancement in the tools available for diagnosis and treatment of such diseases.

## Data Availability

The data that support the findings of this study are available from the corresponding author upon reasonable request.
